# Strength and Surface Characteristics of 3D-Printed Resin Crowns for the Primary Molars

**DOI:** 10.3390/polym15214241

**Published:** 2023-10-27

**Authors:** Soyoung Park, Wontak Cho, Hyeonjong Lee, Jihyeon Bae, Taesung Jeong, Jungbo Huh, Jonghyun Shin

**Affiliations:** 1 Department of Pediatric Dentistry, Dental and Life Science Institute & Dental Research Institute, School of Dentistry, Pusan National University, Yangsan 50612, Republic of Korea; syparkpedo@pusan.ac.kr (S.P.); tsjeong@pusan.ac.kr (T.J.); 2 Department of Prosthodontics, Dental and Life Science Institute & Dental Research Institute, Education and Research Team for Life Science on Dentistry, School of Dentistry, Pusan National University, Yangsan 50612, Republic of Korea; joonetak@hanmail.net (W.C.); say0739@daum.net (J.B.); 3 Department of Prosthodontics, College of Dentistry, Yonsei University, Seoul 03722, Republic of Korea; prostho.hjlee@gmail.com

**Keywords:** compressive strength, crowns, tooth, deciduous, printing, three-dimensional

## Abstract

Some resin polymers available for three-dimensional (3D) printing are slightly elastic, which may be advantageous when used for full crown coverage of the primary teeth. This study was performed to evaluate the mechanical properties of two types of 3D-printed resin crowns in terms of strength and surface characteristics. Polymer resins used for temporary crowns (TCs) and temporary flexible dentures (TFDs) were tested. Digitally designed crowns with different thicknesses (0.4 and 0.6 mm) were 3D-printed. Milled zirconia crowns were used as the control. The static and dynamic fracture loads of the crowns were measured. The crown surface was evaluated using scanning electron microscopy. The average strength did not differ between the types of crowns. The differences between the dynamic and static fracture loads were insignificant. In the TC group, thicker crowns showed lower strength both under static and dynamic loads. After thermomechanical loading, microcracks and dropouts of macrofillers were detected on the surface of all types of resin crowns. The deposition of abraded debris occurred more in the TFD group. The 3D-printed resin crowns were thought to endure biting forces in children. However, some limitations of the material itself should be improved for consideration as a new treatment option in pediatric dentistry.

## 1. Introduction

Dental caries are a common dental problem [1]. The American Academy of Pediatric Dentistry recommends full crown coverage for structural and functional recovery in cases of moderate to severe tooth destruction [2]. Since their introduction in 1950 by Humphrey, preformed metal crowns (PMCs) have been widely used to restore the primary molars. Because of the thin and highly elastic margin of PMCs, clinicians can easily modify the shape of the crown on the prepared tooth. Moreover, a PMC can be passed over the convexity of the primary molar, thus deriving additional retention from the cervical undercut. A simple procedure and relatively favorable prognosis allow for easy application in pediatric patients, but a major drawback is reduced esthetics because of the metallic color [3,4].

Esthetics is an important determinant in selecting materials for caries treatment, even in children [5]. Esthetically satisfactory restorations improve patients’ self-esteem and confidence in achieving smooth interpersonal relationships [6]. To resolve the esthetic drawback of metal crowns, alternatives such as open-faced and pre-veneered stainless-steel crowns have been used, but their prognosis is questionable [3,7]. Compared with the performance of other crowns, preformed zirconia crowns have outstanding esthetics, high strength, and a high level of biocompatibility [8]. However, the time required for tooth preparation varies greatly depending on the skills of the clinician because the crown is so brittle that cannot be modified [9]. Compared with PMCs, zirconia crowns are limited in minimally invasive dentistry because of the extensive tooth reduction required (approximately two-fold anteriorly and 1.8-fold posteriorly) for achieving the passive fit [10].

Elasticity is the most helpful factor for full crown coverage of the primary molars because elastic crowns are expected to bear the stress induced when the crown passes over the small amount of convexity remaining during tooth preparation. With advancements in digital dentistry, computer-aided design and computer-aided manufacturing (CAD/CAM) have been applied across various fields of dentistry. In the CAM method, additive manufacturing commonly known as 3D printing has been tried because of rapid and precise production, with advantages in energy saving [11,12]. Various materials, such as ceramics, metals, and polymers, are suitable for 3D printing. Moreover, it is possible to change some mechanical properties by altering the printing direction, setting the objects straight, tilted, or laid down, despite the design and materials being the same [13]. However, only a few cases trying 3D printing in pediatric dentistry have been reported [14].

Elastic polymers, which are commonly used for temporary flexible dentures, are predicted to be utilized in producing elastic esthetic crowns that can obtain retention from the undercut after successfully passing through convexities without fracturing. In this study, we fabricated 3D-printed resin crowns and evaluated the compressive strength based on the crown thickness and material type. We also assessed the surface properties after an aging simulation and discussed whether the elastic polymer is acceptable for creating strong and esthetically pleasing crowns.

## 2. Materials and Methods

### 2.1. Fabrication of the Crown and Abutment

**Figure 1:**
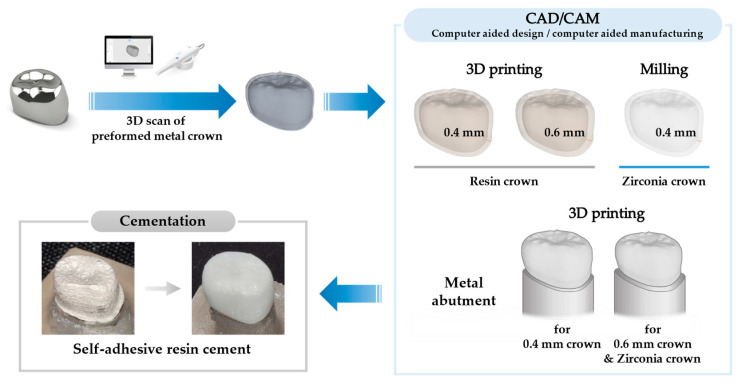
Fabrication procedure for resin crowns and metal abutments.

Figure 1 shows the preparation procedure for the crowns and abutments. The composition of the materials used in this study is listed in Table 1.

The shape of the crown was designed based on commercially available PMCs (I crown, Seil Global, Busan, Republic of Korea) of the mandibular right first primary molar (#84). Using a lab scanner (3shape E3 lab scanner, 3shape, København, Denmark), the exterior of the PMC was scanned, and 3D data were obtained as a standard tessellation language (STL) file. Using CAD/CAM software (Meshmixer v3.5, Autodesk, San Rafael, CA, USA), crowns with different thicknesses were designed. To set the internal surface of the crown, the external surface of the crown offset—0.4 and 0.6 mm—was set. Final crown 3D meshes were fabricated by integrating 2 layers of meshes, original and downsized 3D meshes, with a uniform gap.

Resin for temporary crowns with Ministry of Food and Drug Safety approval (TC-80DP, Graphy, Seoul, Republic of Korea; TC) and proto-version resin for temporary flexible dentures (TFD-23-5, Graphy, Seoul, Republic of Korea; TFD) were used to make resin crowns. The crowns were printed using a digital light processing 3D printer (SprintRay Pro 95, SprintRay Inc., Los Angeles, CA, USA). The uncured resin was washed with isopropyl alcohol, and ultraviolet irradiation was used for final strengthening (CureM U102H, Graphy, Seoul, Republic of Korea).

**Figure 2:**
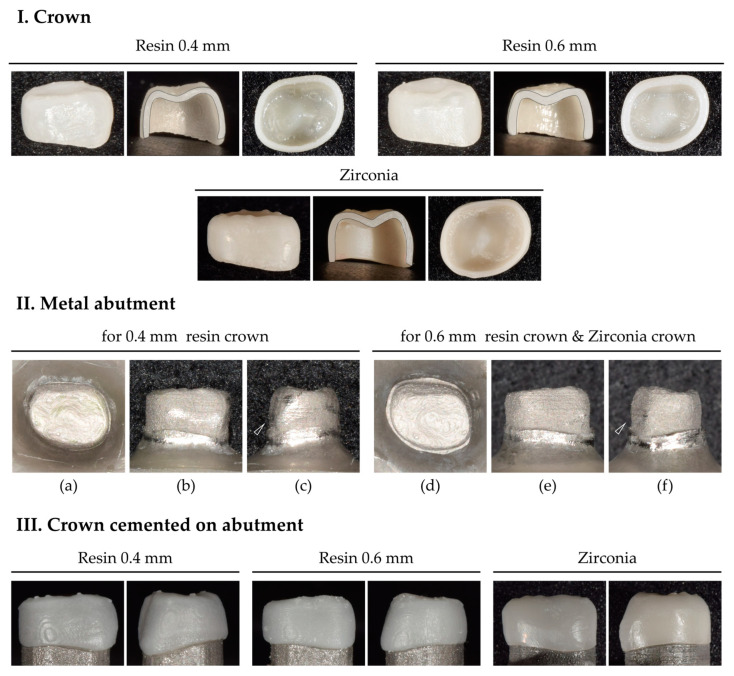
Crown and metal abutment. (**I**) 0.4 and 0.6 mm thick 3D-printed resin crowns and 0.4 mm thick milled zirconia crowns. Sectioned crowns verified that the crowns were designed with uniform thickness in all parts of them. (**II**) 3D-printed metal abutments. Occlusal view (**a**,**d**); buccal view (**b**,**e**); mesial view (**c**,**f**). In the mesial view, the cervical convexity is preserved. (**III**) Crowns cemented to the metal abutment.

Similarly, zirconia crowns were designed with a 0.4 mm thickness and fabricated by milling (MG5D-001, Dental Plus Co., Ltd., Seongnam, Republic of Korea) the zirconia blocks (Luxen Enamel, Dentalmax, Cheonan, Republic of Korea) (Figure 2I).

Downsized 3D meshes, which had been used to set internal surfaces of 0.4 and 0.6 mm for the crowns, were modified to make metal abutments. As luting space was set to 100 µm, downscaled 3D meshes were designed with a 100 µm offset from the crown’s interior surface. The anatomical features, particularly the cervical convexity of the primary first molar, were preserved (Figure 2II(c,f), arrows). Using a cobalt–chromium alloy (CCM-15 metal powder, High Dental Korea, Seoul, Republic of Korea), the abutments were manufactured using a selective laser melting 3D printer (Metal 3D Print Ki 200 P1, Profeta, Nanjing, China) and heat-treated for strengthening (Figure 2II).

### 2.2. Experimental and Control Groups

The experimental groups were classified according to the type of resin polymer (thickness) as follows: TC04 (TC 0.4 mm), TC06 (TC 0.6 mm), TFD04 (TFD 0.4 mm), and TFD06 (TFD 0.6 mm). The zirconia crowns were used as the control group. Each group comprised 20 crowns [15].

### 2.3. Crown Cementation

Before cementation, the metal abutment surface was cleaned by sandblasting it with aluminum oxide powder. Each crown was cemented on the paired abutment. The zirconia crown, which was brittle, was cemented to the abutment for the 0.6 mm crown to obtain a passive fit (Figure 2III). Two-thirds of the crowns were filled with self-adhesive resin cement (RelyX™ U200, 3M ESPE, St Paul, MN, USA). Crowns were fully seated on the metal abutment with a finger press, and excess cement was removed after tack curing. For the initial setting, crowns on abutments were pressed with 5 N of force (using 500 g weight) for 10 min. The final setting was followed by using a light curing unit (Valo^®^, Ultradent, South Jordan, UT, USA) in standard mode (1000 mW/cm^2^) for 20 s. A compressive test was conducted 24 h after crown cementation.

### 2.4. Fatigue Simulation

#### 2.4.1. Preparation of the Antagonist

Fifty primary canines were collected after obtaining approval from the Institutional Review Board, and informed consent was obtained from the patients’ guardians. Teeth were extracted during the treatment of pediatric patients aged 6–12 years visiting our hospital. Teeth with little abrasion of the cusp and no dental caries or restorative materials were selected. Any debris on the surface of the teeth was removed using a prophylaxis brush and a low-speed handpiece. Before testing, the prepared teeth were stored in a sealed container and refrigerated (4 °C) to prevent drying.

#### 2.4.2. Chewing Simulation and Thermocycling

Metal abutments with crowns and the antagonist’s teeth were fixed in an acrylic resin block (Caulk Orthodontic Resin, Dentsply Sirona, Philadelphia, PA, USA) using a 1.0 × 1.0 × 1.0 cm silicone mold (Express™ STD, 3M Dental Product, St. Paul, MN, USA) [16].

**Figure 3:**
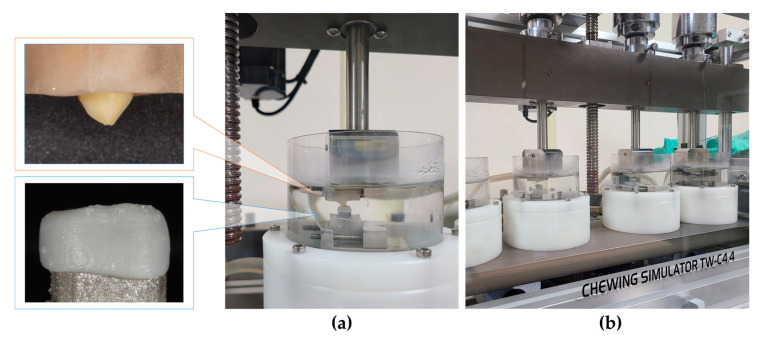
Setup for the thermomechanical loading test. (**a**) Specimens and antagonists fixed in chambers. (**b**) Chewing simulator.

For every 10 teeth in the experimental and control groups, thermomechanical loading tests were performed using a chewing simulator (Dual-Axis Chewing Simulator TW-C4.4, Taewon Tech, Incheon, Korea). The abutments with the crown and antagonist were fastened to the lower and upper parts of each chamber, respectively, and the position was adjusted for occlusion at the central fossa of the crown (Figure 3). According to the standard chewing path, a single chewing cycle was set to 2.0 mm vertical and 0.7 mm lateral motion, and a 5.0 kg load was applied to each chamber for a 50 N chewing force. The loading cycle was applied 120,000 times at 1.6 Hz speed [16]. Simultaneously, thermocycling at 5 and 55 °C was performed with 50 s of fill time and 10 s of drain time.

### 2.5. Field Emission Scanning Electron Microscope (FE-SEM) Analysis

After the chewing simulation, the crown surface was examined independently for intact and abraded areas using FE-SEM (JSM-7200F, JEOL Ltd., Tokyo, Japan). The magnitudes were 100×, 500×, 1000×, and 5000× at 10 kV and 68.80 μA.

### 2.6. Compression Strength and Fracture Pattern Analysis

The compression strength was tested on 10 crowns, each categorized into static and dynamic strengths depending on whether thermomechanical loading was conducted or not. A universal testing machine (3345 Machine, Instron, Norwood, MA, USA) was used to apply the load at a 1 mm/min crosshead speed to the central fossa of the crown with a stainless-steel ball of 3 mm diameter. The strength at the time of the crown fracture was recorded in N [17].

Following the measurement, the crowns were photographed using a digital camera (D3500, Nikon, Tokyo, Japan) and a macro-lens (AF-S Micro NIKKOR 60 mm 1:2.8G ED, Nikon, Tokyo, Japan) to analyze the fracture form.

### 2.7. Statistical Analysis

Statistical analyses were performed using SPSS (version 23.0, IBM Corp., Armonk, NY, USA). Data normality was tested with the Shapiro–Wilk test and the Kolmogorov–Smirnov test. Static and dynamic strengths based on the crown type were analyzed with the Kruskal–Wallis test. Static and dynamic strengths based on the crown thickness were analyzed with a Mann–Whitney U test. The significance level was set at *p* < 0.05.

## 3. Results

### 3.1. Strength

#### 3.1.1. Static Strength

The average static strength of the 0.4 mm thick crowns was 3079.20 ± 955.57 N in the TC group, 3533.92 ± 644.19 N in the TFD group, and 3632.71 ± 596.74 N in the zirconia group, showing no statistically significant difference (*p* = 0.119; Table 2).

The strength according to the crown thickness showed different patterns depending on the type of resin used. In the TC group, the mean strength was 1033.23 ± 717.57 N for TC06 and 3079.2 ± 955.57 N for TC04, showing a significant difference (*p* = 0.000). In the TFD group, the mean strength was 3991.88 ± 443.30 N for TFD06 and 3533.92 ± 644.19 N for TFD04, showing no significant difference (*p* = 0.353; Table 3).

#### 3.1.2. Dynamic Strength

Among the three crown types with the same thickness (0.4 mm), the dynamic strength was slightly higher for TFD04 (3260.17 ± 915.38 N) than for TC04 (2472.78 ± 576.25 N) and the zirconia (2585.75 ± 665.54 N), without statistical significance (*p* = 0.113; Table 2).

Among the crown types of different thicknesses, the mean dynamic strength in the TC group was 1824.10 ± 1346.70 N for TC06 and 2472.78 ± 576.25 N for TC04, showing a significant difference (*p* = 0.035). However, in the TFD group, the mean strength was 3800.15 ± 764.96 N for TFD06 and 3260.17 ± 915.38 N for TFD04, showing no significant difference (*p* = 0.280; Table 3).

Static and dynamic strengths did not differ between the different crown types in thermomechanical loading (*p* = 1, Wilcoxon rank-sum test).

### 3.2. Surface Evaluation

**Figure 4:**
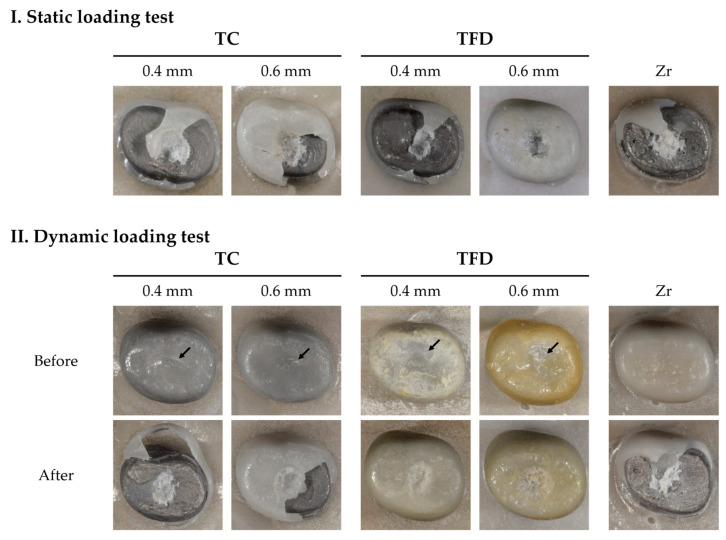
Crowns after the loading test. Static and dynamic fracture loads are categorized depending on whether thermomechanical loading was conducted or not. Resin crowns deformed (pressed) at first and eventually fractured. TFD04 and TFD06 show pressed tendencies rather than fracturing after thermomechanical loading.

After thermomechanical loading, zirconia crowns showed almost no discernible changes. All resin crowns showed an abrasion pattern in the areas of occlusion with the antagonist. Compared with TC, TFD displayed more debris deposition and yellowish discoloration (Figure 4II, TFD). After the compressive loading test, all resin crown types showed an area of partial deformation (compression), while some crowns were fractured. The fracture pattern of the zirconia crowns showed no deformation (Figure 4).

**Figure 5:**
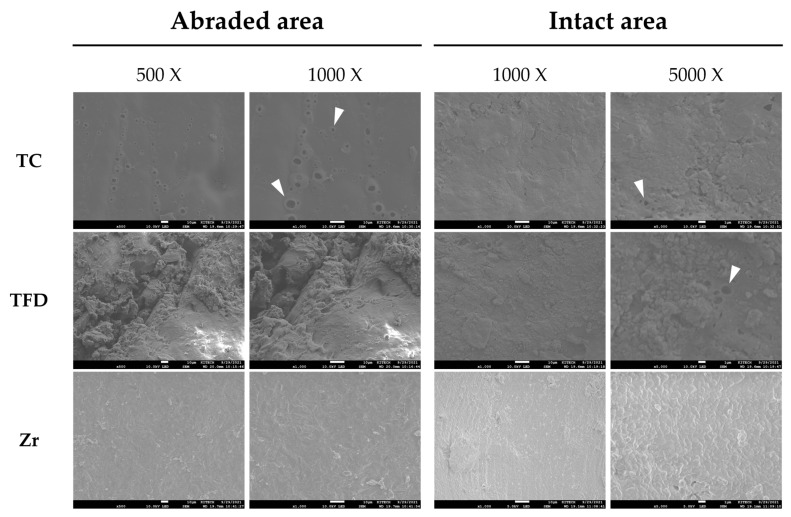
Scanning electron micrographs of the abraded and intact areas of the crown surface after the thermomechanical loading test.

SEM (Figure 5) demonstrated multiple microcracks on the abraded surface in both the TC and TFD groups. TFD displayed more debris deposition compared with TC, while TC had more pits on the surface. The intact areas also exhibited debris deposition and multiple pits in both resin crown types. The zirconia crown had a uniform surface morphology with almost no abrasion or debris deposition on occlusal or occlusal-free areas.

## 4. Discussion

Restorations can be fractured by a single strong force but be more vulnerable to weak but repeated loads. Fatigue is a major cause of failure; therefore, dental restorations should have the mechanical properties to withstand repeated mastication [18]. The thermomechanical cyclic loading test is regarded as a reliable method to test the durability of restorations in clinical practice, as it predicts their failure [19]. Dental caries in the primary molars treated with PMCs are mostly seen in patients under 8 years of age, with a mean age of 4–6 years [4], and the age at which the primary molar exfoliates and the premolar erupts is 10–12 years [20]. For the durability test of crowns in this study, the maximum occlusal force of 3- to 12-year-old children was set as the standard. Bite force in pediatric patients increases with age [21]. The average maximum bite force in the molar regions is 180 N in children aged 3–6 years and 330.5–374.4 N in children aged 6–11 years [21,22]. The range of loading for chewing simulation ranges from 20 to 300 N, while a force not exceeding 50% of the fracture strength of the material is recommended [19,23]. In this study, a 50 N load was applied 120,000 times to simulate 6 months of chewing [23]. The mean bite force of healthy adults without parafunctional habits is approximately 50 N, while that of children and adolescents might be less than 50 N. However, the frequency of parafunctional habits, such as nocturnal bruxism, is high in pediatric patients [24], possibly resulting in the teeth being under a transient force stronger than the mean bite force. Thus, the crown durability in this study was tested at an adequately high level of force.

The compressive strength did not differ significantly according to the crown type (*p* = 0.1, Kruskal–Wallis test) or chewing simulation (*p* = 1, Wilcoxon rank-sum test). The primary mandibular right first molars were targeted in this study. Braun et al. reported the maximum occlusal forces in the primary maxillary first molar and premolar regions, i.e., 78 N in children aged approximately 6 years and 106 N in children aged approximately 10 years [25]. This study showed that the lowest strength of the resin crown was 616.00 N (TC06, dynamic strength), exceeding the maximum occlusal force in children aged 6–10 years. If 3D-printed resin crowns are used to restore the primary molars, the crowns would be expected to endure the masticatory force of children.

When considering the anatomical characteristics of the primary molars, TFD resin was determined to be a more reliable material for fabricating crowns, and a 0.4 mm thick crown is thought to exhibit advantages with respect to tooth preservation. Contrary to the TFD group, the average static and dynamic strengths were higher for TC04 than for TC06. This could be attributed to the inherent properties of the resin materials. The compositions of TC and TFD were identical, except for the presence of butylhydroxytoluene (Table 1). Both materials belong to the polymethyl methacrylate group, and adding butylhydroxytoluene makes the material more brittle and reduces elasticity [26]. In the case of brittle materials, internal defects exert a significant influence on the strength. In this study, the strength of TC06 was lower than that of TC04; a similar pattern was reported in a recent study using 3D-printable resin [27]. This is presumably because invisible microcracks occurred more readily, owing to the reduced deformation tendency and increased stress. Crowns fabricated using TC resin could be advantageous when the tooth preparation form allows for a passive fit. Crowns fabricated using TFD resin, which is more elastic compared with TC, were more likely to better withstand the stress generated by passing the cervical convexity. The amount of tooth preparation might be reduced like in cases of PMC restoration.

As 3D printing allows us to use various materials and rapidly fabricate complex forms [28], it is expected to be widely utilized in dentistry. Stereolithography and digital light processing, which are widely applied printing methods in dentistry, use light irradiation for polymerization. During processing, the platform moves a certain distance upward, layer by layer, and the liquid resin flows into the space between the platform and the reservoir [29]. Therefore, 3D-printing resins require special fluidity. With the increase in viscosity, the perfection of the products decreases because of gravity and surface tension. Lower viscosity is advantageous for 3D printing [30]. Viscosity is related to the amount of filler. However, reducing the total amount of filler to maintain fluidity is challenging because adding fillers is inevitable in order to improve mechanical properties. Macrofillers have been used as alternatives by reducing the total surface area to obtain good mechanical properties and low viscosity [29].

After the thermomechanical loading test, the resin crowns exhibited discoloration and surface debris deposition. Pits and cracks, presumed to be traces of filler loss, were verified with SEM (Figure 5, white arrows). In a resin complex, the interface between the filler and the substrate can serve as a passage for water. In addition to hydration, temperature change caused by thermocycling causes the materials to repeatedly expand and contract, thereby causing filler loss on the surface [31]. In this case, discoloration was detected more easily because the escape of macrofillers made the surface rougher, which influenced the scattering of light, leading to a perceived color change. In contrast, SEM showed that the zirconia crowns had clean surfaces with almost no abrasion or debris deposition. Preformed zirconia crowns exhibit outstanding surface smoothness with almost no plaque deposition and little irritation of the gingival tissue [15]. As surface roughness is associated with discoloration and the deposition of foreign substances, improvements in minimizing surface changes should be considered for application to pediatric patients considering oral hygiene and aesthetics.

The abrasion of the resin crown surface caused by the antagonist after the chewing simulation was visually examined (Figure 4II, black arrow). Particles removed from the crown or tooth can act as abrasives at the interface between the tooth and the restorative material, increasing the rate of abrasion via the three-body wear mechanism [32]. As aforementioned, because large fillers might fall out more easily, the surface of the 3D-printed resin crown is predicted to undergo easier abrasion upon chewing, which is likely to affect the abrasion of the antagonist. In pediatric patients, the abrasion of the crown or antagonist is clinically important, as it might change the occlusion; therefore, a quantitative analysis of abrasion should be conducted to provide supporting evidence.

To date, the use of 3D printing has been recommended to be limited to a medium-term period not exceeding 24 months because of the problem of strength related to filler content [29]. Therefore, 3D printing has been utilized for temporary prosthetics, such as onlays, crowns, and bridges. For the primary molars, the expected usage span of crown restorations is 4–8 years [4,20]. Owing to the process of 3D printing, the fluidity of resin polymers is critical, and increased filler size is inevitable [29]. In this study, surface characteristics were associated with problems of color stability, debris deposition, and abrasion resistance, which implied clinical limitations for the esthetics and durability of restorations. Nevertheless, it is inspiring that 3D printable materials for permanent restoration have started to be marketed [33]. For the clinical application of 3D-printed resin crowns, studies need to be underway on the improvement of materials and post-manufacturing modifications, such as surface treatments.

## 5. Conclusions

Considering the maximum bite force of children and adolescents, 3D-printed resin crowns have acceptable strength when considering a treatment option for pediatric patients. Based on the cyclic loading test, crowns fabricated using TFD resin were more advantageous. Also, some limitations were found with respect to color and surface changes. If the materials and 3D printing processes were continuously improved, the production of esthetic resin crowns with elasticity might be realized by using the 3D printing technique.

## Figures and Tables

**Table 1:** Raw materials used for crown fabrication.

Type	Abbreviation	Product (Lot No.)	Composition	Content (%)	Flexural Strength (MPa)	Flexural Modulus (MPa)	Manufacturer
3D-printed polymer	TC	TC-80DP (B1220K11-003)	α,α′-[(1-Methylethylidene)di-4,1-phenylene]bis[ω-[(2-methyl-1-oxo-2-propenyl)oxy]poly(oxy-1,2-ethanediyl)	0–20	≥220	≥4500	Graphy, Seoul, Korea
			1,4-Butanediol polymer with α-hydro-ω-hydroxypoly(oxy-1,4-butanediyl) and 1,1′-methylendbis [4-isocyanatobenzene]	20–60			
			2-Hydroxyethyl methacrylate	30–80			
			Phenylbis(2,4,6-trimethylbenzoyl)phosphine oxide	0–10			
			Butylhydroxytoluene	0–1			
			Titanium dioxide	0–1			
			Pigment	0–1			
	TFD	TFD-23-5 (prototype)	α,α′-[(1-Methylethylidene)di-4,1-phenylene]bis[ω-[(2-methyl-1-oxo-2-propenyl)oxy]poly(oxy-1,2-ethanediyl)	0–20	≥100	≥2850	Graphy, Seoul, Korea
			1,4-Butanediol polymer with α-hydro-ω-hydroxypoly(oxy-1,4-butanediyl) and 1,1′-methylendbis[4-isocyanatobenzene]	20–60			
			2-Hydroxyethyl methacrylate	30–80			
			Phenylbis(2,4,6-trimethylbenzoyl)phosphine oxide	0–10			
			Titanium dioxide	0–1			
			Pigment	0–1			
Zirconia	Zr	Luxen Enamel (201113-10E2P-7)	ZrO_2_ + HfO_2_ + Y_2_O_3_	>99.8 wt.%			Dentalmax, Cheonan, Korea
			(Y_2_O_3)_	(4–6 wt.%)			

**Table 2:** The compression strength of crowns fabricated using different materials.

	Static Fracture Load (N)					Dynamic Fracture Load (N)				
	n	Mean	SD	Max	Min	n	Mean	SD	Max	Min
TC04	10	3079.20	955.57	4435.58	2152.53	10	2472.78	576.25	3265.73	1537.46
TFD04	10	3533.92	644.19	4440.78	2958.33	10	3260.17	915.38	4469.83	2065.35
Zr	10	3632.71	596.74	4435.97	2643.25	10	2585.75	665.54	3632.56	1823.38
*p*	0.119					0.113				

Kruskal–Wallis test; N, Newton; n, number of samples; SD, standard deviation; Max, maximum; Min, minimum.

**Table 3:** The compression strength of crowns with different thicknesses.

	Static Fracture Load (N)					Dynamic Fracture Load (N)				
	n	Mean	SD	Max	Min	n	Mean	SD	Max	Min
TC04	10	3079.20	955.57	4435.58	2152.53	10	2472.78	576.25	3265.73	1537.46
TC06	10	1333.23	717.57	2994.81	764.78	10	1824.10	1346.70	4428.30	616.00
*p*	0.000					0.035				
TFD04	10	3533.92	644.19	4440.78	2958.33	10	3260.17	915.38	4469.83	2065.35
TFD06	10	3991.88	443.30	4405.87	2891.68	10	3800.15	764.96	4566.61	2224.63
*p*	0.353					0.280				

Mann–Whitney U test; N, Newton; n, number of samples; SD, standard deviation; Max, maximum; Min, minimum.

## Data Availability

The data presented in this study are available on request from the corresponding author.
